# Short-term effects of sugar-free apricot jam, cocoa powder and dried cranberry cereal bar on glycaemic responses in healthy adults: a randomised clinical trial

**DOI:** 10.1017/jns.2022.74

**Published:** 2022-09-14

**Authors:** Emilia Papakonstantinou, Emmanuella Magriplis, George Katsaros, Dimitra Glinou, Manolis Sofiadis, Virginia Skoulidi, Antonis Zampelas

**Affiliations:** 1 Laboratory of Dietetics and Quality of Life, Department of Food Science and Human Nutrition, School of Food and Nutritional Sciences, Agricultural University of Athens, Athens, Greece; 2 Institute of Technology of Agricultural Products, Hellenic Agricultural Organization ‘DEMETER’, Athens, Greece

**Keywords:** Cereal bar, Cocoa, Glycaemic index, Glycaemic responses, Jam

## Abstract

High sugar intake has been associated with adverse effects on health, with some types of breakfast being highly linked to overweight and obesity. The aim was to compare the effects of four sugar-free breakfast items, apricot jam with white bread (JWB), white bread (WB), cocoa with fat-free milk (CM), and dried cranberry cereal bar (CB), compared to d-glucose on the glycaemic responses. Using a cross-over design, twelve healthy individuals (25 ± 4 years; BMI 22 ± 2 kg/m^2^) received isoglucidic test meals (25 g of available carbohydrate) and 25 g glucose reference, in random order. Glycaemic index/load (GI/GL) were calculated, and capillary blood glucose samples were collected at 0–120 min after meal consumption. Subjective appetite was assessed with visual analogue scales. Sugar-free apricot jam and cocoa powder contained traces of available carbohydrates and were consumed along with bread and fat-free milk, respectively. JWB and WB were classified as medium GI, low-to-medium GL; CM as medium GI, low GL; and CB as high GI, low-to-medium GL. Subjective hunger was lower after JWB, fullness was higher after CM and pleasure was higher after CB (*P* for all < 0⋅05). In conclusion, sugar-free apricot jam with and without WB and cocoa powder with fat-free milk are suitable healthy breakfast options leading to improved glycaemic and subjective appetite responses.

## Graphical abstract

**Figure:**
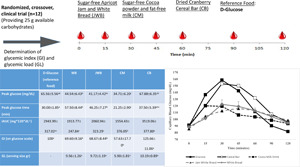

## Introduction

Consumption of free sugars has been linked to dental caries, overweight and obesity, type 2 diabetes, cardiovascular diseases and non-alcoholic fatty liver disease^(1–5)^. Sucrose (table sugar) belongs to the class of simple carbohydrates and is widely used in the food industry as the main compound for sweetness. The World Health Organization (WHO) has issued guidance on the intake of sugars, recommending that intake be significantly reduced^(5)^. The European Food Safety Authority (EFSA) recently concluded that the intake of added and free sugars should be as low as possible in the context of a nutritionally adequate diet as the available data do not allow the setting of a science-based Upper Intake Level (UL) or a safe level of intake for either total, added or free sugars^(6)^. The EFSA experts explained that this is because the risk of adverse health effects (responses) increased across the whole range of observed intake levels (doses) in a constant (linear) manner, i.e., the higher the intake, the greater the risk of adverse effects^(6)^. For these reasons, public health interventions aim to reduce the consumption of foods rich in simple sugars^(7)^. It is reasonable to assume that the interest in using low and no-calorie sweeteners as possible alternatives to sugar has been intensified. Sweeteners are commonly used as sugar substitutes by the food industry because they can either not be metabolised or provide negligible amounts of energy^(8)^. Sweeteners are generally divided into two categories, the non-caloric high-intensity sweeteners, which are substances with an intense sweet taste but without energy value (i.e., steviol glycosides, aspartame, sucralose), and the nutritive sweeteners, typically polyols (i.e., sorbitol, maltitol, xylitol) containing approximately half the calories of sugar^(9)^. These alternatives in addition to producing lower postprandial glucose excursions and responses, impart lower calories compared to that of sugar^(10)^. Results from a recently published systematic review and meta-analysis of randomised controlled clinical (RTC) trials examining the health effects of non-sugar sweeteners confirmed that consuming low/no-calorie sweeteners leads to a significant reduction in energy intake, primarily when compared to sugars and may be effective in assisting with short-term weight loss, without affecting overall glucose control or other cardiometabolic risk factors^(4)^. On the other hand, low certainty evidence from observational studies should be interpreted with caution as residual confounding and reverse causality may partially or significantly influence the observed associations, which should be addressed in future studies^(4)^.

The glycaemic index (GI) is a tool developed to classify carbohydrate-containing foods according to their time-integrated effects on postprandial glycaemia^(11,12)^. The GI depicts both the standardised and relative postprandial glucose response based on an equal amount of available carbohydrate and relative to a referent food^(13)^. Foods containing carbohydrate that is digested, absorbed and metabolised quickly are considered high GI foods (GI ≥ 70 on the glucose scale) whereas those that are digested, absorbed and metabolised slowly are considered low GI foods (GI ≤ 55 on the glucose scale)^(13)^. The glycaemic load (GL) is the product of GI and the total available carbohydrate content in a given amount of food^(13)^. Consumption of high GI foods is associated with increased chronic disease risk^(13–15)^; whereas low-to-medium GI foods are considered favourable to health^(11)^. A moderate improvement in glycaemic control may be accomplished when low GI foods replace higher GI foods^(16)^.

The aim of this study was to evaluate the short-term effects of sugar-free typical breakfast items, namely an apricot jam, a dried cranberry cereal bar and cocoa powder on GI, GL, postprandial glycaemic responses and subjective appetite.

## Materials and methods

### Subjects

Twelve healthy subjects (six men, six women), between 18 and 50 years, were recruited by a variety of methods, including notices posted around the university campus and online advertisement. Using the *t* distribution and assuming an average CV of within-individual variation of incremental AUC (iAUC) values of 25 %, *n* 10 participants have 80 % power to detect a 33 % difference in iAUC with two-tailed *P* < 0⋅05. In the present investigation, we enrolled and studied twelve participants. Participants underwent an initial screening. Measurements included anthropometry (height, body weight, waist and hip circumferences), body composition analysis via bioimpedance method (InBody 230, Biospace, USA), and fasting blood glucose via finger prick (calibrated MediSmart® Ruby glucose meter with lancing device, Lilly-PHARMASERV SA, Greece). Additionally, a questionnaire on general health was completed. Subjects were non-smokers, had a healthy body mass index (BMI between 18⋅5 and 24⋅9 kg/m^2^), a normal BP (systolic pressure <120 mmHg and a diastolic pressure <80 mmHg), normal fasting blood glucose concentration via finger prick (<5·6 mmol/l), and no medical conditions (i.e., cardiovascular diseases, diabetes mellitus, liver diseases, nephropathy, clinical depression, gastrointestinal disorders), nor taking medications known to affect glycaemia (glucocorticoids, metformin, thyroid hormones, thiazide diuretics), and were not allergic to the test foods. Also, women were not pregnant/lactating nor were they diagnosed with polycystic ovary syndrome. All twelve participants completed all treatments and were included for analysis.

The study was conducted at the Laboratory of Dietetics and Quality of Life, Agricultural University of Athens, Greece. All subjects gave their informed consent for inclusion before participating in the study. The study was conducted in accordance with the Declaration of Helsinki, and the protocol was approved by the Bioethics Committee of the Agricultural University of Athens (EIDE Reference Number 23). This trial was registered at Clinicaltrials.gov (NCT04857554).

### Study design

Three food products, typical breakfast choices, an apricot jam, cocoa powder and a dried cranberry cereal bar with no added sugars were produced (Yiotis, S.A., Attica, Greece). Appropriate sugar alternatives were used to obtain low-sugar, low-fat foods similar to that of their sugar counterparts. For this purpose, sugar alternatives having physical properties closer to those of sugar, such as sorbitol in combination with steviol glycosides or sucralose and/or maltitol, were used. The glycaemic indexes (GIs) of sugar-free foods were evaluated. The GI was determined according to ISO 26642:2010 International Organization for Standardization^(12)^ method and procedures. The study consisted of six dietary treatments in a randomised, open-label type, cross-over design: two glucose reference drinks, and the four test meals tested once: a white bread (WB); a white bread and apricot jam (JWB); a cocoa with fat-free milk drink (CM) and a cereal bar with cranberries (CB). Eligible participants were studied on six separate days over a period of 3–9 weeks with an interval of no less than 40 h and more than 2 weeks between tests. Participants attended six test sessions of about 3 h, separated by a wash-out period of at least 2 d. Online computer software (Social Psychology Network, Middletown, CT, USA) was used for simple randomisation of the sequence of the test foods (http://www.randomizer.org)^(17)^. A researcher not involved in the collection and analysis of the scientific data was responsible for the randomisation of the volunteers to the intervention days examining the test foods. Participants arrived at the test centre around 8:45–9:00 h in the morning following an overnight fast of 10–14 h. Participants were asked to maintain stable dietary and activity habits throughout their participation in the study. In addition, participants were instructed to refrain from alcohol on the previous evening, from vigorous exercise on the morning of the test, and were only allowed to eat the provided foods throughout the test sessions. If any participant was not feeling well or had not complied with the preceding experimental conditions, the test was not carried out and was rescheduled for another day. On each test occasion, participants were weighed. Each session consisted of a test food that had to be consumed at a comfortable pace within 12–15 min and 2 h post-consumption measurement of metabolic blood parameters. Participants were instructed to consume the glucose drink at a comfortable pace within 10 min. Participants received in random order the reference food (d-glucose), tested two times (i.e., first, sixth visit), and the four test meals: WB, JWB, CM and CB, tested once, in different weeks, with a random sequence in accordance to the recommended GI methodology^(12)^. All the test foods and the reference foods were given in portions containing 25 g of available carbohydrates (54⋅6 g WB (120 kcal); 50 g apricot jam (73 kcal) with 52⋅4 g WB (120 kcal); 36 g cocoa (104 kcal) with 500 ml fat-free milk drink (187⋅5 kcal); and 74 g cranberries cereal bar (259 kcal). The portion of 25 g of available carbohydrates was chosen according to ISO 26642:2010 International Organization for Standardization^(12)^ because the portion size of apricot jam and cocoa powder providing 50 g of glycaemic carbohydrate was unreasonably large (>1⋅00 kg) to consume. Test meals were served with 250 ml of water as a drink in all six trials.

### Test meals

During each of the six test sessions, participants consumed one of the following test meals: a commercial WB; a commercial WB with apricot jam (JWB), made with sugar substitutes (sorbitol, steviol glycosides, apricot); a cocoa with fat-free milk (CM), 32 % cocoa, made with 80 % sugar substitutes (maltitol, sucralose and steviol glycosites) and a dried cranberries cereal bar (CB), (cereals 28⋅6 % (rice, maize flour), made with sugar substitutes (maltitol, maltitol syrup, isomaltitol, steviol glycosides), and oat flour (Yotis S.A., Attica, Greece); or glucose reference drink (25 g anhydrous glucose dissolved in 250 mL water). The available carbohydrates were determined according to the method AOAC 991.43 with the Megazyme assay kit (Megazyme kit-K-ACHDF, Megazyme Ltd, Scotland, UK), which calculates only the carbohydrates that can be absorbed (sugars and digestible starch), neglecting dietary fibre and resistant starch (RS). The nutritional characteristics of the studied test products according to their food label are described in Table 1 and the available carbohydrates in Table 2. The tested sugar-free apricot jam and cocoa test foods contained minimal amounts of available carbohydrates (0⋅41 g and 0⋅83 g/serving of food, respectively). For this reason, the apricot jam was consumed with WB and cocoa powder with fat-free milk to achieve the target of 25 g of available carbohydrates. The available carbohydrates % wet basis was apricot jam: 2⋅03; cocoa powder: 4⋅16; dried cranberries cereal bar: 33⋅82. The available carbohydrates (g)/per portion of food was 20 g apricot jam: 0⋅41; 20 g cocoa powder: 0⋅83; 24 g cereal bar: 8⋅11; 30 g WB: 13⋅74; 250 ml fat-free milk: 9⋅4; 20 g cocoa with 200 ml fat-free milk: 10⋅23; 20 g apricot jam with 30 g WB: 14⋅15. When calculating the portion sizes, we took into account that 25 % of sorbitol and 40 % of maltitol are considered as glycaemic/available carbohydrates.

**Table 1.** Macronutrient composition per 100 g described based on the food label

	Apricot jam	Cocoa powder	Cereal bar
Energy (kJ/kcal)	543/156	1205/289	1424/339
Fat (g)	0⋅2	5⋅5	5⋅7
Saturated fat (g)	62	3⋅3	1⋅2
Carbohydrates (g)	2⋅9	74	73
Sugar (g)	2⋅9	1⋅2	0⋅4
Glucose (g)	1⋅0	3⋅8	32⋅3
Fructose (g)	1⋅0	0⋅4	1⋅5
Polyols (g)	58	64	23
Starch	0	7⋅3	50
Dietary fibre (g)	0	10	6⋅6
Protein (g)	0⋅2	5⋅2	4⋅7
Salt (g)	0⋅02	0⋅31	0⋅30

Portion sizes recommended on the food label are: 20 g cocoa powder (served with 200 ml fat-free milk), 24 g dried cranberries cereal bar, 20 g apricot jam.

**Table 2.** Baseline participants’ characteristics

	Total (*n* 12)
*N*	12 (6 men, 6 women)
Age (years)	24⋅83 ± 3⋅79
Body weight (kg)	63⋅90 ± 11⋅44
Height (cm)	168⋅69 ± 8⋅00
Body mass index (BMI; kg/m^2^)	22⋅33 ± 2⋅39
Basal metabolic rate (BMR; kcal)	1505⋅03 ± 221⋅66
Body fat (kg)	14⋅36 ± 3⋅55
Fat-free mass (kg)	27⋅43 ± 7⋅19
Waist circumference (cm)	74⋅75 ± 8⋅70
Hip circumference (cm)	95⋅25 ± 8⋅51
Dietary intake (from 24 h recall)	
Energy intake (kcal)	1616⋅80 ± 622⋅83
Carbohydrates (g)	245⋅30 ± 98⋅00
Total fat (g)	120⋅00 ± 58⋅50
Protein (g)	97⋅40 ± 28⋅10
Sodium (mg)	1988⋅40 ± 119⋅78

Values are means ± sem.

### Blood glucose concentrations

On each test occasion, three fasting blood samples were obtained by finger-stick at 5 min intervals (−5, 0); the average of the glucose concentrations at these two time points was taken to be the baseline (fasting) concentration. Then participants were served a test food together with 250 ml water. They were instructed to consume all of the food and water at a comfortable pace within 12–15 min. Further finger-prick blood samples were collected at 15, 30, 45, 60, 90 and 120 min after starting to eat. Each blood glucose time value was the mean of three blood samples from the same drop of blood of each participant. Before and during the test, a blood glucose test record was filled out with the participant's initials, identification number, date, body weight, test food, beverage, time of starting to eat, time it took to eat, time and composition of last meal and any unusual activities. During the 2 h test, participants remained seated quietly. After the last blood sample had been obtained, participants were offered a snack and then allowed to leave. To standardise all data collection procedures, capillary blood glucose monitoring was performed at the fingertip (distal phalange of the third finger). Blood glucose was measured with calibrated glucometers using glucose dehydrogenase-FAD test strips (Ruby Blood glucose Test Strips, Lilly-PHARMASELV S.A., Athens, Greece), which show no reactivity to any sugars other than glucose and have better heat resistance and oxygen resistance. We chose to determine the GI of test foods using a calibrated glucometer, as it has been adequately reported that GI results obtained by glucometers are reliable and similar to those attained by enzymatic kits methods^(18–20)^. The repeatability and within laboratory coefficient variations were 3⋅4 %. The average blood glucose response curve was plotted by calculating the mean blood glucose concentrations of all participants at each time point (Fig. 2). Then for each sample and each study subject, the incremental area under the curve (iAUC) was calculated geometrically, using the trapezoid rule, and ignoring the area beneath the baseline^(12)^. The GI calculation for each test food sample used the method referred to as the mean of the ratios. For each subject, the ratio between the individual iAUC after consuming the test food sample and the iAUC for the same subject after consuming the reference food was calculated and expressed as a percentage value. Then, the GI of each test food was calculated as the average value of the ratios across all the subjects consuming the test food samples. Peak blood glucose, defined as the highest recorded glucose value minus the baseline value, and peak blood glucose time, defined as the time elapsing from the start of a meal to the highest recorded glucose value were calculated.

### Subjective appetite

Subjects rated their hunger, desire to eat and perceived fullness after eating on 100 mm line visual analogue scales (VAS), ranging from not at all (0 mm) to extremely (100 mm), with for example not hungry (0 mm), full (100 mm) or having desire for food in the middle (50 mm). VAS were given in the form of a booklet, one scale per page^(21)^. VAS ratings were obtained at times 0, 15, 30, 45, 60, 90 and 120 min post-test meal consumption.

### Dietary intake

Dietary intake was assessed by 24 h recalls at every visit and analysed using the Diet Analysis Plus program, as well as using Hellenic and European Food Composition Databases (http://www.eurofir.org/foodinformation/foodcomposition-databases-2/). The databases were modified to include new foods and recipes. The purpose of collecting dietary intake was to confirm that participants refrained from changing their eating habits until the study was completed.

### Statistical analysis

Data are presented as mean ± standard error of the mean (sem), unless otherwise specified. Statistical analysis was based on data distribution tested by kernel density plots. Differences in baseline continuous variables were evaluated using analysis of variance (ANOVA) for normally distributed continuous variables, Kruskal–Wallis test for skewed continuous data and Pearson's chi-square test for categorical variables. Data were entered into a spreadsheet by two different individuals and the values were compared to assure accurate transcription. Incremental areas under the glucose curves (AUC), ignoring area below fasting, were calculated. For the purposes of the AUC calculation, fasting glucose was taken to be mean of the first measurement of the blood glucose concentrations at times −5 min, and 0 min. The GI was calculated by expressing each participant's AUC for the test food as a percentage of the same participant's mean AUC for the three d-glucose drinks controls. If values were found to have >2 sd above the mean, they would be excluded. No outlying GI values were found. The ISO method requires, for a valid GI measurement, that the mean within-individual coefficient of variation of glycaemic responses elicited by repeated tests of oral glucose (termed reference CV) is ≤30 %^(12)^. We calculated reference CV for glucose according to the ISO method; namely the mean, sd and CV (100 × sd/mean) of the glucose iAUC values elicited by the two repeated tests of 25 g glucose were calculated for each participant and the mean of the resulting values was the reference CV. The blood glucose concentrations at each time, AUC, GI values, subjective appetite and BP were subjected to repeated-measures ANOVA examining for the main effects of the test food and the food × participant interaction. Between treatments, ANOVA for a 2 × 2 cross-over study was conducted for blood glucose. In a 2 × 2 design, we assume that there are no individual effects since a complete randomisation process was followed for treatment allocation. The models included the factors ‘subject’ (id), ‘sequence’ for inter-subject variation, and ‘period’ & ‘treatments’ to account for intra-subject variability. Time × test meal interaction was evaluated. Multiple comparisons between the interventions were tested *post hoc* using the Tukey test with Bonferroni correction. For all other parameters, one-way ANOVA was used to investigate differences between test meals followed by *post hoc* Tukey test and Bonferroni correction. Differences in blood pressure were assessed with Paired T-test analysis. Differences in VAS ratings were evaluated using one-way ANOVA and the Friedman's test. Correlations between GI and test meals’ characteristics were determined with Spearman's rho coefficient. Means differing by more than the LSD (least significant difference) were statistically significant, two-tailed *P* < 0⋅05. GL was calculated using the formula: GL = GI × g of available carbohydrate in a typical food serving/100. Data were analysed using SPSS 20.0 software (SPSS Inc., Chicago, IL, USA).

## Results

### Subjects’ baseline characteristics

The subjects’ characteristics can be found in Table 2. There were no intermittent missing values of dropouts.

### GI of the tested meals

The results of GI and GL for the three tested sugar-free breakfast items are presented in Table 3. The results revealed different GI values for the three tested samples (Table 3). According to the present classification^(12)^, sugar-free apricot jam and cocoa powder are classified as low GI foods (GI ≤55 on glucose scale); WB, apricot jam with white bread (JWB) and cocoa with fat-free milk drink were classified as medium GI (GI> 55 but <70 on glucose scale) foods; and dried cranberries cereal bar was classified as high GI (GI ≥70 on glucose scale) food. The results also revealed that all sugar-free tested products: apricot jam, cocoa powder, apricot JWB, cocoa with fat-free milk drink and dried cranberries cereal bar were classified as low GL foods (GL ≤10 per size portion).

**Table 3.** Incremental area under the curve (iAUC) for capillary blood glucose, peak glucose, peak time for glucose, GI and GL for the test meals relative to the reference d-glucose values

	d-Glucose (reference food)	WB	JWB	CM	CB
Peak glucose (mmol/l)	3·64 ± 0·31^ab^	2·47 ± 0·36^b^	2·29 ± 0·25^bc^	1·93 ± 0·34^c^	3·77 ± 0·35^ab^
Peak glucose time (min)	30⋅00 ± 1⋅85^a^	57⋅50 ± 8⋅44^b^	46⋅25 ± 7⋅27^b^	21⋅25 ± 2⋅90^c^	37⋅50 ± 5⋅39^abc^
iAUC (mmol*120*L^−1^)	163·56 ± 17·61^ac^	106·32 ± 13·77^c^	114·50 ± 17·96^c^	86·36 ± 20·89^b^	195·50 ± 20·99^a^
GI (on glucose scale)	100^a^	70 ± 9^b^	69 ± 8^b^	58 ± 18^b^	126 ± 11^a^
GL (serving size (g))	−	10 ± 1^a^	10 ± 1^a^	6 ± 2^a^	10 ± 1^a^

WB: white bread; JWB: apricot jam and white bread; CM: cocoa and fat-free milk drink; CB: cereal bar with dried cranberries. Values are Mean ± sem. Glycaemic index (GI) and Glycaemic Load (GL) were calculated by the FAO/WHO method. Each value represents the mean of twelve participants.

Values marked with the same superscript letter are not significantly different (*P* > 0⋅05). Means were compared column-wise by using one-way ANOVA for factor ‘treatment’, period and sequence of treatment and *post hoc* Tukey test with Bonferroni correction to account for multiple comparisons between test meals; *P*-values < 0⋅05 were considered as significant.

### Glycaemic responses to sugar-free tested products

**Fig. 1.**
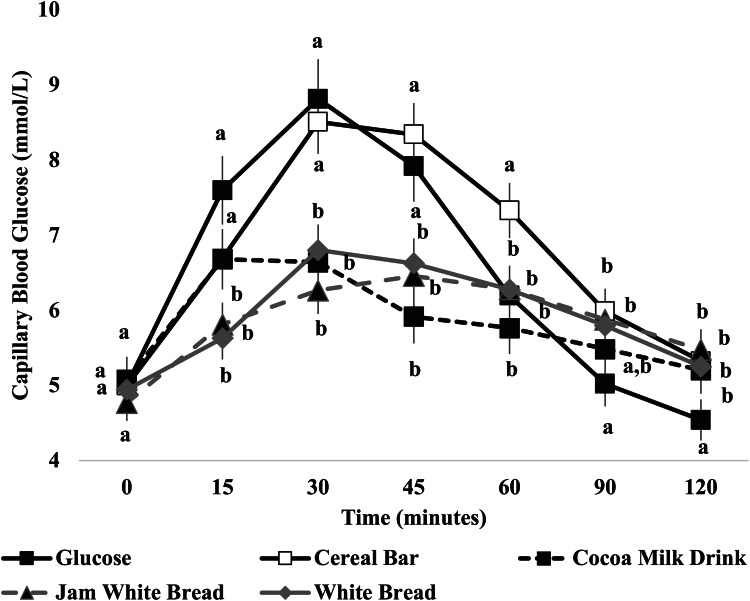
Glycaemic responses after consumption of d-glucose (reference food) and the test meals, dried cranberries cereal bar, cocoa with fat-free milk drink, apricot jam with white bread, and white bread (*n* 12). Data are means ± sem. Data were compared by *post hoc* analysis of repeated-measures ANOVA. The models included the factors ‘subject’ (id), ‘sequence’ for inter-subject variation, and ‘period’ & ‘treatments’ to account for intra-subject variability.

The change in postprandial glucose over time (120 min) can be seen in Fig. 1. No significant differences were observed in fasting glucose concentrations between glucose and the test breads (*P* for all > 0⋅05; Fig. 1). There was a significant blood glucose × time interaction (*P* < 0⋅001), a blood glucose × time × test meal interaction (*P* < 0⋅001) and a time × test food interaction (*P* < 0⋅001). There was a main effect of the test meal on blood glucose concentrations (*P* < 0⋅001). Compared to the reference food (d-glucose), significantly lower blood glucose concentrations were observed only after the consumption of JWB and WB at 15 min (*P* < 0⋅001 and *P* < 0⋅001, respectively; Fig. 1), without significant differences between JWB and WB (*P* > 0⋅05; Fig. 1). Compared to the reference food (d-glucose), significantly lower blood glucose concentrations at 30 min were observed after the consumption of JWB (*P* < 0⋅001), WB (*P* < 0⋅001) and CM (*P* < 0⋅001) respectively; Fig. 1), without significant differences between and among JWB, WB and CM (*P* > 0⋅05; Fig. 1). Compared to the reference food (d-glucose), lower blood glucose concentrations at 45 min were observed for JWB (*P* = 0⋅006), WB (*P* = 0⋅015) and CM (*P* < 0⋅001); Fig. 1), without significant differences between and among JWB, WB and CM (*P* for all > 0⋅05; Fig. 1). Compared to the reference food (d-glucose), significantly higher blood glucose concentrations were observed at 60 min only for CB (*P* = 0⋅002; Fig. 1). Compared to the reference food (d-glucose), significantly higher blood glucose concentrations at 90 min were observed for CB (*P* = 0⋅004), JWB (*P* = 0⋅010) and WB (*P* = 0⋅018; Fig. 1); without significant differences between and among CB, JWB and WB (*P* for all > 0⋅05; Fig. 1). Compared to the reference food (d-glucose), significantly higher blood glucose concentrations at 120 min were observed for CB (*P* = 0⋅025), JWB (*P* = 0⋅007) and WB (*P* = 0⋅044; Fig. 1); without significant differences between and among CB, JWB and WB (*P* for all > 0⋅05; Fig. 1).

Compared to CB, significantly lower blood glucose concentrations at 15 min were observed for JWB (*P* = 0⋅028) and WB (*P* = 0⋅009); without significant differences between JWB and WB (*P* > 0⋅05, Fig. 1). Compared to CB, significantly lower blood glucose concentrations at 30 min were observed for JWB (*P* < 0⋅001), WB (*P* < 0⋅001) and CM (*P* < 0⋅001; Fig. 1), without differences between and among JWB, WB and CM (*P* > 0⋅05; Fig. 1). Compared to CB, significantly lower blood glucose concentrations at 45 min were observed for JWB (*P* < 0⋅001), WB (*P* = 0⋅001) and CM (*P* < 0⋅001; Fig. 1), without differences between and among JWB, WB and CM (*P* > 0⋅05; Fig. 1). Compared to CB, significantly lower blood glucose concentrations at 60 min were observed for JWB (*P* = 0⋅004), WB (*P* = 0⋅004) and CM (*P* < 0⋅001; Fig. 1), without significant differences between and among JWB, WB and CM (*P* > 0⋅05; Fig. 1).

There was a main effect of test meal on peak glucose values (*P* < 0⋅001). Compared to the reference food (d-glucose), JWB and CM had significantly lower peak blood glucose values (JWB: *P* = 0⋅046, CM: *P* < 0⋅04, respectively; Table 3) without significant differences between JWB and CM (*P* > 0⋅05; Table 3). Compared to CB, JWB and CM have significantly lower peak glucose values (JWB: *P* = 0⋅020, CM: *P* = 0⋅002, respectively; Table 3). There was no difference regarding peak glucose values between JWB and WB (*P* > 0⋅05; Table 3). There was a main effect of test meal on peak time for glucose (*P* < 0⋅001). Compared to the reference food (d-glucose), only WB has a significantly larger peak time for glucose (*P* = 0⋅013; Table 3). CM had a significantly lower peak time for glucose compared to JBW (*P* = 0⋅032) and WB (*P* < 0⋅001; Table 3); without significant differences with CB and the reference food (d-glucose) (*P* for all >0⋅05; Table 3).

There was a significant main effect of the test meal on 0–120 min iAUC for blood glucose (*P* < 0⋅001; Table 3). The mean within-participant variation of 0–120 min iAUC for blood glucose for the repeated tests was 22⋅5 %. Compared to the reference food (d-glucose), the 0–120 min iAUC for blood glucose values calculated only for CM was significantly lower (*P* = 0⋅045; Table 3). Compared to CB, the 0–120 min iAUC for blood glucose values calculated were lower for JWB (*P* = 0⋅030), WB (*P* = 0⋅012) and CM (*P* = 0⋅001; Table 3); without significant differences between and among JWB, WB and CM (*P* for all >0⋅05; Table 3).

### Subjective appetite assessment

**Fig. 2.**
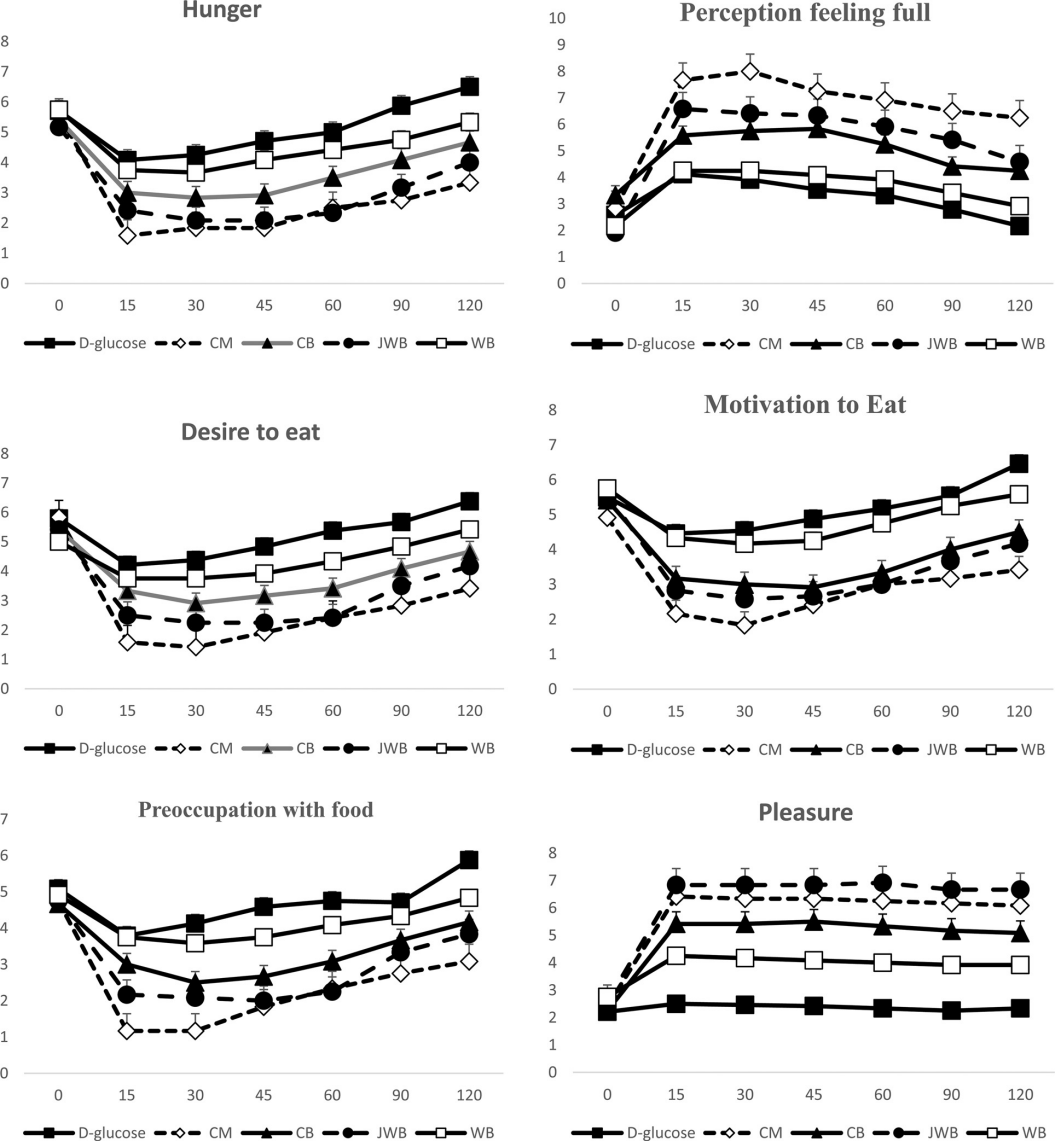
Subjective appetite ratings from visual analog scales (*N* 12). Data are means ± sem.

Results on subjective appetite assessment are shown in Fig. 2. There was a main effect of test meal on iAUC for hunger (*P* = 0⋅025). Compared to the reference food (d-glucose), significantly lower mean iAUC for hunger was observed for CB (*P* = 0⋅042) and JWB (*P* = 0⋅042). There was a main effect of test meal on mean total hunger 0–120 min (*P* = 0⋅011). There were no significant differences for hunger between test meals at baseline (*P* > 0⋅05; Fig. 2). Compared to the reference food (d-glucose), only CM had a significantly lower mean total hunger 0–120 min (*P* = 0⋅017). Compared to the reference food (d-glucose), hunger at 15, 30, 45, 60, 90 and 120 min was significantly lower for CM (*P* = 0⋅003, *P* = 0⋅003, *P* = 0⋅001, *P* = 0⋅005, *P* = 0⋅001 and *P* = 0⋅002, respectively; Fig. 2) and for JWB (*P* = 0⋅039, *P* = 0⋅008, *P* = 0⋅002, *P* = 0⋅003, *P* = 0⋅005 and *P* = 0⋅014, respectively; Fig. 2). Compared to the reference food (d-glucose), hunger at 45 min was significantly lower for CB (*P* = 0⋅029; Fig. 2). Compared to WB, significantly lower hunger was observed for JWB at 30, 45 and 60 min (*P* = 0⋅048, *P* = 0⋅015 and *P* = 0⋅018, respectively; Fig. 2).

There was a main effect of test meal on the mean total desire for food 0–120 min (*P* = 0⋅016). There were no significant differences for the desire for food between test meals at baseline (*P* > 0⋅05; Fig. 2). Compared to the reference food (d-glucose), significantly lower mean total desire for food 0–120 min was observed only for CM (*P* = 0⋅015; Fig. 2). Compared to the reference food (d-glucose), a significantly lower desire for food was observed at 15 for CM (*P* = 0⋅003; Fig. 2) and tended to be lower for JWB (*P* = 0⋅051). Compared to the reference food (d-glucose), significantly lower desire for food was observed at 30, 45, 60, 90 and 120 min for CM (*P* = 0⋅001, *P* = 0⋅001, *P* = 0⋅002, *P* = 0⋅005, and *P* = 0⋅004, respectively; Fig. 2) and for JWB (*P* = 0⋅010, *P* = 0⋅003, *P* = 0⋅002, *P* = 0⋅028, and *P* = 0⋅031, respectively; Fig. 2). Compared to WB, significantly lower desire for food was observed for JWB at 45 and 60 min (*P* = 0⋅048 and *P* = 0⋅042, respectively; Fig. 2).

There was a main effect of test meal on iAUC for preoccupation with food (*P* = 0⋅044). There was a main effect of test meal on mean total preoccupation with food 0–120 min (*P* = 0⋅030). Compared to the reference food (d-glucose), significantly lower mean total preoccupation with food 0–120 min was observed for CM (*P* = 0⋅036). There were no significant differences for preoccupation with food between test meals at baseline (*P* > 0⋅05; Fig. 2). Compared to the reference food (d-glucose), a significantly lower preoccupation with food at 15 min was observed for CM (*P* = 0⋅003; Fig. 2) and tended to be lower for JWB (*P* = 0⋅055). Compared with the reference food (d-glucose), a significantly lower preoccupation with food at 30, 45, 60 and 120 min was observed for CM (*P* = 0⋅001, *P* = 0⋅002, *P* = 0⋅012 and *P* = 0⋅011, respectively; Fig. 2). Compared with the reference food (d-glucose), significantly lower preoccupation with food at 30, 45 and 60 min was observed for JWB (*P* = 0⋅018, *P* = 0⋅004, *P* = 0⋅009, respectively; Fig. 2). Compared with the reference food (d-glucose) significantly lower preoccupation with food at 45 min was observed for CB (*P* = 0⋅029; Fig. 2). Compared to WB, significantly lower preoccupation with food was observed for JWB at 45 min (*P* = 0⋅046), with a tendency to be lower at 60 min (*P* = 0⋅053).

There was a main effect of test meal on iAUC for the perception of feeling full (*P* = 0⋅002). Compared to the reference food (d-glucose), a significantly higher iAUC for perception of feeling full was observed only for JWB (*P* = 0⋅001). Compared to WB and CB, higher iAUC for the perception of feeling full was observed for JWB (*P* = 0⋅026 and *P* = 0⋅041, respectively). There was a main effect of test meal on the mean total perception of feeling full 0–120 min (*P* < 0⋅001). Compared to the reference food (d-glucose), a significantly higher mean total perception of feeling full 0–120 min was observed for CM (*P* < 0⋅001). There were no significant differences for the perception of feeling full for food between test meals at baseline (*P* > 0⋅05; Fig. 2). Compared with the reference food (d-glucose) significantly higher perception of feeling full at 15 min was observed for CM (*P* < 0⋅001; Fig. 2) and JWB (*P* = 0⋅002; Fig. 2). Compared with the reference food (d-glucose) significantly higher perception of feeling full was observed at 30, 45, 60 min for CM (*P* < 0⋅001, *P* < 0⋅001 and *P* < 0⋅001, respectively; Fig. 2), JWB (*P* = 0⋅002, *P* = 0⋅001 and *P* = 0⋅002, respectively), and CB (*P* = 0⋅018, *P* = 0⋅007 and *P* = 0⋅020, respectively; Fig. 2). Compared to the reference food (d-glucose), significantly higher perception of feeling full was observed at 90 and 120 min for CM (*P* < 0⋅001 and *P* < 0⋅001, respectively) and JWB (*P* = 0⋅004 and *P* = 0⋅012, respectively; Fig. 2).

There was a main effect of the test meal on iAUC for a total amount of food that one could consume at that specific moment named ‘motivation to eat’ (*P* = 0⋅037). There was a main effect of test meal on mean total motivation to eat 0–120 min (*P* = 0⋅007). Compared to the reference food (d-glucose), significantly lower meal total motivation to eat 0–120 min was observed only for CM (*P* = 0⋅014). There were no significant differences for motivation to eat between test meals at baseline (*P* > 0⋅05; Fig. 2). Compared to the reference food (d-glucose), significantly lower motivation to eat at 15 min was observed for CM (*P* = 0⋅003) and JWB (*P* = 0⋅033). Compared to the reference food (d-glucose), significantly lower motivation to eat at 30, 45 and 60 min was observed for CM (*P* < 0⋅001, *P* = 0⋅001 and *P* = 0⋅009, respectively; Fig. 2), JWB (*P* = 0⋅009, *P* = 0⋅003 and *P* = 0⋅009, respectively; Fig. 2) and CB (*P* = 0⋅036, *P* = 0⋅008 and *P* = 0⋅027, respectively; Fig. 2). Compared to the reference food (d-glucose), significantly lower motivation to eat at 90 min was observed for CM (*P* = 0⋅006; Fig. 2) and JWB (*P* = 0⋅028; Fig. 2). Compared to the reference food (d-glucose), significantly lower motivation to eat at 120 min was observed for CM (*P* = 0⋅001), JWB (*P* = 0⋅010) and CB (*P* = 0⋅027) (Fig. 2). Compared to WB, lower motivation to eat was observed for JWB at 15, 30, 45 and 60 min (*P* = 0⋅049, *P* = 0⋅032, *P* = 0⋅029 and *P* = 0⋅034, respectively; Fig. 2).

There was a main effect of test meal on iAUC for pleasure for food (*P* = 0⋅040). Compared to the reference food (d-glucose), only JWB had significantly higher iAUC for pleasure for food (*P* = 0⋅047). There was a main effect of test meal on mean total pleasure for food 0–120 min (*P* < 0⋅001). Compared to the reference food (d-glucose), higher total mean pleasure for food 0–120 min was observed for CM (*P* < 0⋅001), JWB (*P* < 0⋅001) and CB (*P* = 0⋅027). There were no significant differences for pleasure for food between test meals at baseline (*P* > 0⋅05; Fig. 2). Compared to the reference food (d-glucose), significantly higher pleasure for food was observed at 15, 30, 45, 60, 90 and 120 min for CM (*P* < 0⋅001, *P* < 0⋅001, <0⋅001, *P* < 0⋅001, *P* < 0⋅001 and *P* < 0⋅001, respectively; Fig. 2), JWB (*P* < 0⋅001, *P* < 0⋅001, *P* < 0⋅001, *P* < 0⋅001, *P* < 0⋅001 and *P* < 0⋅001, respectively; Fig. 2) and CB (*P* = 0⋅002, *P* = 0⋅001, *P* = 0⋅001, *P* = 0⋅001, *P* = 0⋅001 and *P* = 0⋅002, respectively; Fig. 2). Compared with the reference food (d-glucose), significantly higher pleasure for food was observed for WB at 90 min (*P* = 0⋅044; Fig. 2), with a tendency to be higher at 15 min (*P* = 0⋅051), at 30 min (*P* = 0⋅057), at 45 min (*P* = 0⋅059), at 60 min (*P* = 0⋅056) and at 120 min (*P* = 0⋅068). Compared to WB, higher pleasure for food was observed for JWB at 15, 30, 45, 60, 90 and 120 min (*P* = 0⋅005, *P* = 0⋅004, *P* = 0⋅002, *P* = 0⋅001, *P* = 0⋅001 and *P* = 0⋅002, respectively; Fig. 2). No differences between and among test meals were observed for thirst, expected consumption and portion of consumption (*P* for all > 0⋅05; Fig. 2).

## Discussion

This study showed that the sugar-free apricot jam and cocoa powder contained minimal amounts of available carbohydrates, less than the 10 g available carbohydrates per serving as a prerequisite for GI testing and were classified as low carbohydrate foods. Adding the sugar-free apricot jam to WB did not increase postprandial glucose responses, and WB consumed alone or with jam produced similar postprandial glycaemic responses. Adding the cocoa powder to fat-free milk produced higher than anticipated GI, but a low GL, and a significantly lower postmeal blood glucose compared to d-glucose. Both the addition of jam to WB and cocoa powder to fat-free milk lowered hunger, desire to eat, motivation to eat, preoccupation with food and produced a higher perception of feeling full and pleasure for food. Moreover, removing sugar from the dried cranberry cereal bar produced a high GI, but low GL food, and also resulted in lower perceived hunger.

The use of GI for the classification of carbohydrate-rich foods has been endorsed by the FAO/WHO, who recommended that the GI of foods should be considered together with information about food composition to guide food choices^(11)^. Consumption of foods and meals that induce a lower glycaemic response and delay gastric emptying, thus leading to decreased insulin requirements and postprandial glucose excursions, has been proposed as an important strategy to ameliorate postprandial hyperglycaemia and insulin resistance^(22)^. Such foods typically contain low amounts of easily absorbable carbohydrates^(22–24)^. Lowering the GI of a food may be significant as it has been shown that consumption of low GI foods may be sufficient to achieve a lower glycaemic response from one meal to the next^(25–28)^. In cohort studies, the GL, but not the carbohydrate content, has been frequently linked to reduced risk of type 2 diabetes^(29)^ and cardiovascular diseases^(15)^. It has been suggested that the GL is a good predictor of the postprandial glycaemic responses to a particular food^(30)^. It has also been shown that lowering the GL of consumed carbohydrates leads to a significant haemoglobin A1C reduction of –0⋅2 to –0⋅5 %^(31,32)^.

The food industry is using several low or no caloric sweeteners in their food products in an effort to reduce sugar content. Sorbitol has been shown to cause delayed gastric emptying, decrease glucose absorption^(33)^, increase muscle glucose uptake and inhibit intestinal glucose absorption^(34)^, and is classified as a low GI compound (GI = 9). Maltitol has been ascribed values ranging from 25 to 75^(35)^, likely because the total weight of maltitol has been assumed to be available, which is not the case (only 40 % is)^(12)^. Although it has been shown to inhibit small intestinal glucose absorption^(36)^ and most have classified it as a low GI compound (GI = 35)^(37)^, due to its high available carbohydrate content it is likely to fall in the high GI category and should be further investigated. Steviol glycosides and sucralose, both no-calorie sweeteners do not interfere with glucose homeostasis^(10)^.

The food industry produces various fruit products such as jams and fruit spreads with similar textures. The GI and GL values of jam diversify from those of fruit spread and marmalade due to the different manufacturing processes^(38–40)^. It has been shown that the sugar or sugar replacers content changes the GI value of a jam^(40)^. Dried apricots have been shown to mitigate postprandial glycaemic responses and to be negatively correlated to GI^(41)^. One study found that a jam with a 52⋅6 % content ratio of maltose and glucose from maize syrup and glucose syrup was classified as a high GI (76), whereas a jam with a 49⋅4 % content ratio of fructose from apple juice concentrate or with polydextrose as the sugar substitute was classified as a low GI (47 and 17, respectively)^(40)^. One Italian study showed that an apricot jam with apple juice was classified as medium GI food (63) and high GL food (42) with similar results reported for fruit spread with thick apricot (GI = 63 and GL = 41)^(39)^. Another study comparing jams made with sucrose or polydextrose, reported statistically significant differences between GI values of jam with sucrose (GI = 61) and jam with polydextrose (GI = 9)^(40)^. In the present investigation, the sugar-free apricot jam made with sorbitol and steviol glycosides contained minimal amounts of available carbohydrates and subjects would need to consume a non-realistically high amount (1⋅25 kg) of apricot jam in order to obtain 25 g of available carbohydrates required for GI testing. For this purpose, we decided to examine its effects consumed along with WB. The results of the present investigation showing no effect of the apricot jam when added to WB on postprandial glycaemic responses in agreement with others^(40)^. In particular, one study investigated the influence on the postprandial blood glucose response in eight healthy adults after the intake of only 20 g jam and of one slice of bread with 20 g jam^(40)^. Postmeal blood glucose responses of a jam with very low or no sucrose content, but with fructose, glucose and sorbitol did not rise rapidly, the GI obtained was low, and an influence on the postprandial glucose level was barely confirmed^(40)^, similarly to results from the present investigation.

There is a variety of cocoa powder products in the market. The GI value of cocoa powder is classified as low^(42)^, and only one Australian cocoa powder product was classified as high GI and reported to increase postprandial insulin concentrations in healthy adults^(43)^. One study examining the effects of a chocolate drink with aspartame compared to a chocolate drink with sucrose on the glycaemic responses found higher postprandial glucose concentrations after consumption of the beverage containing sucrose compared to sweetener, without differences between the two regarding insulin concentrations^(44)^. Subjects in the present investigation consumed a sugar-free cocoa drink with sweeteners (maltitol, sucralose and steviol glycosides) along with fat-free milk. Fat-free milk is classified as a low GI (GI = 32) food^(42)^. Unlike other studies, the results of the present investigation showed that our cocoa drink was classified as medium GI (GI = 58), but low GL (GL = 6). The low GL produced by our tested product is in agreement with others^(38)^. Apart from the addition of low and no-calorie sweeteners in our tested product, cocoa is a product particularly rich in flavonoids. The primary flavonoids in cocoa are the flavan-3-ols (catechin and epicatechin and proanthocyanidins). Meta-analyses of acute or short-term RTC trials showed that cocoa powder reduced fasting and postprandial insulin concentrations and improved insulin resistance^(45)^. Others have shown that cocoa powder has a direct effect on enzymes and receptors (i.e., a-glycosidase, a-amylase, SGLT-1) involved in glucose metabolism reducing their expression and secretion leading to ameliorated postprandial glycaemic responses achieved in the long term^(45,46)^. Moreover, the ameliorated glycaemic responses observed in the present investigation may be due to the dual effects of cocoa powder and fat-free milk. Fat-free milk has been shown to have a beneficial role in insulin secretion and glycaemic control due to its rich content in essential amino acids and branched-chain amino acids^(47)^. Milk proteins have insulinotropic properties, with the whey fraction being a more efficient insulin secretagogue than casein^(47)^. It remains to be shown whether the insulinotropic effect of whey and milk depends on an optimal and rapid postprandial release of certain amino acids to the blood, the release of a bioactive peptide, or an activation of the incretin system, particularly by enhancing GIP secretion^(48)^.

Fruit cereal bars are popular and convenient snacks. The GI and GL values of cereal bars vary depending on their composition, their production methods, and thus cereal bars have been classified as low, medium or high GI and GL products^(35,49)^. The GI of cereal bars may be affected by many factors, including wheat variety, flour characteristics and processing, the presence of intact cereal grains, presence of fruits, starch interactions with other food components, amylose to amylopectin ratios, addition of viscous fibres, protein digestibility, types of baking processes, the type of sugars and starch and starch digestibility^(50)^. The results of the present investigation found that a sugar-free dried cranberry cereal bar was high GI (GI ≥70), but low GL (GL = 10), because for GI testing in order to achieve 25 g of available carbohydrates we gave to our subjects to consume three cereal bars at one sitting. The present results are in agreement with a study classifying fruit cereal bars as high GI foods^(51)^, but in contrast with another study finding fruit cereal bars as high GL^(35)^. The present results are in agreement with another study investigating cereal bars with different berries, including dried cranberries, showing no effects for cranberry bars regarding glucose and glucoregulatory hormones, nor were there any treatment effects for any berry type regarding ex vivo oxidation, appetite-mediating hormones or appetite^(52)^. One study examining the effect of RS type 4 on postprandial glycaemic responses of cereal bars showed no significant differences between a cereal bar containing no RS4 and two cereal bars with RS4^(53)^. Moreover, another study examining the effects of varying sucrose, RS, and whey protein content of cereal bars on postprandial glucose and insulin responses, showed that inclusion of RS in cereal bar formulations did not reduce glycaemic responses, while higher protein content was associated with a lower glucose iAUC and a higher insulin-to-glucose iAUC ratio^(54)^.

Regarding the VAS, the literature is inconsistent. A glucostatic hypothesis proposed for the regulation of food intake suggested that high glucose concentrations in blood produce satiety^(55)^. Intake of rapidly digested carbohydrates reduces hunger and increases satisfaction in the short-term, and when blood glucose levels start to decrease, hunger increases and the feeling of satisfaction decreases^(56)^. Moreover, high GI carbohydrates have been shown to suppress food intake short-term (<2 h) after consumption^(57)^, while the satiating effect of low GI carbohydrates appears later (>2 h), producing lower postprandial glucose and insulin responses and reducing appetite^(58)^. In addition, the taste of sweetness in products such as jams, cocoa powder beverages and fruit cereal bars, is an important factor that may influence satiety and appetite^(59)^. The latter explain the lower hunger and higher pleasure observed after consumption of the dried cranberry cereal bar tested in the present investigation. The present results are in partial disagreement with a study finding higher satisfaction after consumption of a jam containing sugar and producing higher glucose and insulin responses, but not after sugar-free jams^(60)^. In the present investigation, we reported short-term lower hunger, desire to eat, preoccupation with food, motivation to eat, and higher perception of feeling full and pleasure after consumption of the sugar-free apricot jam taken together with WB, which may be due to the acute satiating effects of both the sugar-free jam and the WB. The present results regarding lower hunger, desire to eat, preoccupation with food, motivation to eat and pleasure after consumption of the cocoa powder with fat-free milk disagree with two other studies reporting no effects of cocoa beverages on satiety^(61,62)^.

### Study limitations and advantages

The strength of our study includes the randomised, cross-over design where each subject served as his own control. The major limitation of the present investigations, as with all acute feeding trials, is the inability to translate these acute findings to long-term benefits. Another shortcoming is the sample size. While the use of twelve participants has been validated by a number of studies, nevertheless this sample size reduces the study precision and may lead to exaggerated associations. Moreover, blood collections from the participants would enable measurements of plasma insulin and incretins.

## Conclusion

In conclusion, the results of this study showed that the tested sugar-free apricot jam and cocoa powder contained minimal amounts of available carbohydrates making them optimal products for consumption from high health risk consumers, such as obese individuals and people with glucose intolerance or diabetes mellitus. The sugar-free typical breakfast foods examined in the present investigation produced different GI values with apricot jam consumed along with WB being classified as medium GI food, cocoa powder consumed along with fat-free milk being classified as medium GI food, and dried cranberry cereal bar being classified as high GI food. Adding the sugar-free apricot jam to WB did not increase postprandial glycaemic responses and adding the cocoa powder to fat-free milk produced lower postprandial glucose responses compared to glucose. Both lowered hunger, desire to eat, motivation to eat, preoccupation with food and produced a higher perception of feeling full and pleasure for food. All three sugar-free products were classified as low GL foods, making them appropriate food choices for long-term glycaemic control.
